# *Bacillus velezensis* SQR9-Emitted Volatiles Enhance *Arabidopsis* Salt Tolerance via ROS Scavenging and Ion Transport Regulation

**DOI:** 10.3390/plants15020218

**Published:** 2026-01-10

**Authors:** Yucong Li, Liming Xia, Yanqiong Meng, Xinyu Shen, Xiang Wan, Fangqun Gan, Ruifu Zhang

**Affiliations:** 1 College of Environment and Ecology, Jiangsu Open University, Nanjing 210036, China; 2 Agricultural Mechanization and Engineering Research Institute, Anhui Academy of Agricultural Sciences, Hefei 230001, China; 3 School of Forestry and Landscape Architecture, Anhui Agricultural University, Hefei 230036, China; 4 School of Chemical Engineering and Materials, Changzhou Institute of Technology, Changzhou 213032, China; 5 College of Resources and Environmental Sciences, Nanjing Agricultural University, Nanjing 210095, China

**Keywords:** *Bacillus velezensis* SQR9, volatile organic compounds (VOCs), salt stress, *Arabidopsis thaliana*, systemic priming, 2,3-butanediol, ROS homeostasis, ion homeostasis, SOS pathway

## Abstract

Salinity stress severely limits crop productivity worldwide. While plant growth-promoting rhizobacteria (PGPR) are known to alleviate abiotic stress, the specific mechanisms mediated by their volatile organic compounds (VOCs) remain largely elusive. In this study, an in vitro split-plate system was used to investigate the effects of VOCs emitted by *Bacillus velezensis* SQR9 on *Arabidopsis thaliana* seedlings under salt stress. Exposure to SQR9 VOCs significantly enhanced *Arabidopsis* salt tolerance, evidenced by increased biomass and root growth. Mechanistically, SQR9 VOCs mitigated salt-induced damage by increasing chlorophyll content, modulating osmolytes, and reducing malondialdehyde (MDA) levels. SQR9 VOCs alleviated oxidative stress by decreasing ROS (H_2_O_2_, O_2_^−^) accumulation and enhancing antioxidant enzyme (SOD, CAT, POD) activities. Furthermore, SQR9 VOCs maintained ion homeostasis by significantly reducing leaf Na^+^ accumulation, maintaining a high K^+^/Na^+^ ratio, and upregulating key ion transporter genes. Analysis of the headspace from SQR9 cultured on MSgg medium identified 2,3-butanediol (2,3-BD) as a major active VOC. Exogenous application of 2,3-BD successfully mimicked the growth-promoting and salt-tolerance-enhancing effects of SQR9. Our findings demonstrate that SQR9 VOCs, particularly 2,3-BD, systemically prime *Arabidopsis* for salt tolerance by co-activating the antioxidant defense system and the SOS ion homeostasis pathway.

## 1. Introduction

Soil salinization is a critical and escalating environmental constraint that severely threatens global agricultural productivity and food security [1,2,3]. Exacerbated by climate change and unsustainable irrigation practices, high salinity affects vast areas of arable land, leading to substantial crop yield losses [4]. The detrimental effects of salinity on plants are twofold: first, it imposes an osmotic stress that limits water uptake, and second, it causes ion toxicity, primarily from the over-accumulation of sodium (Na^+^) and chloride (Cl^−^) ions, which severely disrupts the intracellular K^+^/Na^+^ ratio [5]. This primary imbalance rapidly triggers a secondary oxidative stress, characterized by the uncontrolled production of reactive oxygen species (ROS), such as hydrogen peroxide (H_2_O_2_) and superoxide anions (O_2_^−^), which cause extensive damage to membranes, proteins, and nucleic acids [6,7].

To survive under saline conditions, plants have evolved two major, coordinated defense pillars. The first pillar is the maintenance of ROS homeostasis, which is achieved by a sophisticated antioxidant defense system. This system includes non-enzymatic molecules, such as proline and soluble sugars, which act as osmolytes and radical scavengers, and a battery of enzymatic scavengers, including superoxide dismutase (SOD), catalase (CAT), and various peroxidases (PODs), that work in concert to neutralize excess ROS [8,9,10,11]. The second, equally vital pillar is the maintenance of ion homeostasis. This involves minimizing cytosolic Na^+^ concentration by restricting Na^+^ uptake and actively removing Na^+^ from the cytoplasm. This process is centrally governed by the Salt Overly Sensitive (SOS) pathway (encoded by *SOS1*, *SOS2*, and *SOS3*), which activates the SOS1 plasma membrane Na^+^/H^+^ antiporter to export Na^+^ out of the cell [12,13]. This is complemented by tonoplast-localized Na^+^/H^+^ antiporters (e.g., NHX1) that sequester Na^+^ into the vacuole, and transporters like HKT1 that mediate Na^+^ recirculation within the plant [14,15,16].

The urgent need for sustainable agricultural solutions has highlighted the potential of plant growth-promoting rhizobacteria (PGPR) as an eco-friendly strategy to enhance plant resilience to abiotic stress [17]. Numerous PGPR strains from diverse genera have been shown to promote plant growth and enhance salt tolerance in various crops. The mechanisms underlying PGPR-mediated salt tolerance are diverse, commonly involving the production of osmolytes, improved nutrient acquisition, modulation of plant hormone levels (e.g., IAA, ABA, and ACC deaminase activity), and enhancement of the plant’s antioxidant defenses [18,19,20,21]. However, most studies have focused on mechanisms requiring direct bacteria–plant contact, such as root colonization, biofilm formation, or the secretion of non-volatile compounds (e.g., exopolysaccharides, signal peptides) into the rhizosphere [22,23].

In addition to these direct interactions, PGPR can also communicate with plants over a distance via the emission of volatile organic compounds (VOCs) [24]. The role of bacterial VOCs is well-established in promoting plant growth [25,26,27,28] and in activating induced systemic resistance (ISR) against biotic stressors, such as pathogens and insects [29]. *Bacillus velezensis* SQR9, widely recognized as a model biofertilizer strain, possesses diverse mechanisms to enhance root development and nutrient uptake, and has been specifically identified to alleviate salt stress in plants [30,31]. A foundational study on this strain attributed its salt-tolerance-inducing ability to the secretion of a non-volatile polyamine, spermidine. That study reported that spermidine activated both the plant’s glutathione (GSH) antioxidant pathway and the *SOS*/*NHX* ion homeostasis pathways, but that SQR9-emitted VOCs were ineffective [32].

Despite the clear role of spermidine, the potential for bacterial VOCs to mediate abiotic stress tolerance, particularly salinity, remains a comparatively nascent field [33,34]. A few pioneering studies have suggested that VOCs from other bacterial strains can modulate plant ion homeostasis—for instance, VOCs from *B. amyloliquefaciens* FZB42 were shown to induce *NHX1* and *HKT1* via JA signaling [35], and VOCs from *Alcaligenes faecalis* modulated auxin and gibberellin pathways [36]. However, a comprehensive understanding of how bacterial VOCs systemically prime plants to maintain ion homeostasis under high salinity is still lacking, particularly regarding the identification of the major bioactive compounds involved.

Previous genomic and metabolic analyses have established that *B. velezensis* SQR9 possesses the capacity to produce various volatile compounds, including 2,3-butanediol (2,3-BD) [37,38]. However, whether this specific volatile contributes to the strain’s ability to induce salt tolerance remains to be functionally validated. Therefore, the present study was designed to re-evaluate the volatile-mediated capabilities of *B. velezensis* SQR9, hypothesizing that under specific metabolically conducive culture conditions, its VOCs could systemically prime *Arabidopsis thaliana* for salt tolerance. We aimed to dissect the underlying mechanisms, focusing on the dual pillars of ROS homeostasis and *SOS*-mediated ion homeostasis, and to identify the key effector VOC(s) responsible for this non-contact protective effect, with particular emphasis on validating 2,3-BD as one of the main active volatile compounds produced by this strain.

## 2. Results

### 2.1. Enhancement of Arabidopsis Growth and Salt Tolerance

**Figure 1:**
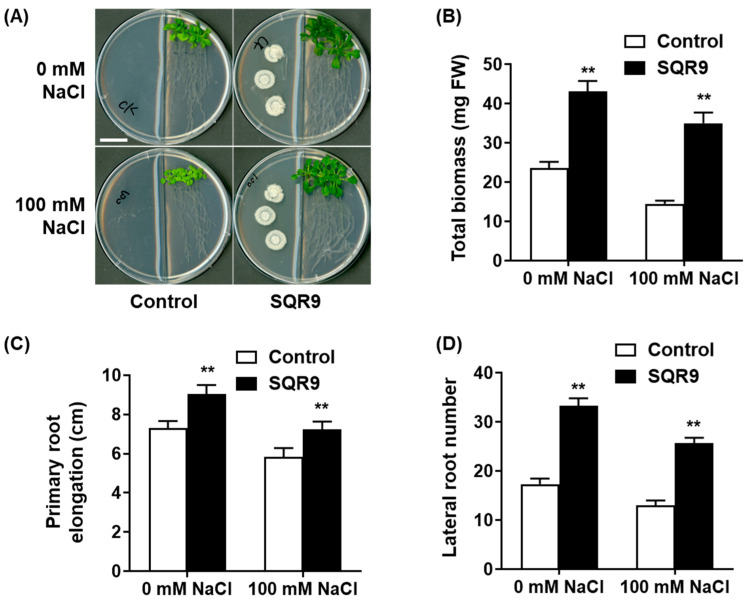
Effect of SQR9-emitted VOCs on *Arabidopsis* growth and salt tolerance. The figure displays the phenotypic and quantitative analysis of growth parameters in 10-day-old *Arabidopsis* seedlings co-cultured with *B. velezensis* SQR9 in a split-plate system, under non-saline (0 mM NaCl) and salt-stressed (100 mM NaCl) conditions. (**A**) Phenotypic analysis of seedlings co-cultured with SQR9 or sterile medium (Control). The scale bar represents 2 cm. (**B**) Average total biomass (mg Fresh Weight, FW). (**C**) Average primary root elongation (cm). (**D**) Average lateral root number. All quantitative data are presented as mean ± standard deviation (SD). Double asterisks (**) indicate a highly significant difference (*p* < 0.01) compared to the Control group under the corresponding NaCl concentration.

To investigate the non-contact, volatile-mediated effect of *B. velezensis* SQR9 on plant growth and salt tolerance, an in vitro split-plate system was established. Under non-saline (0 mM NaCl) conditions, 10-day-old *Arabidopsis* seedlings co-cultured with SQR9 VOCs displayed a significant enhancement in growth compared to the sterile medium control (Figure 1A). This growth-promoting effect was quantified as an 82.4% increase in total biomass (Figure 1B), a 23.7% increase in primary root elongation (Figure 1C), and a 1.9-fold increase in the number of lateral roots (Figure 1D). When subjected to high salt stress (100 mM NaCl), the growth of control seedlings was severely inhibited. These seedlings exhibited pronounced stress symptoms, including chlorotic leaves and severely stunted root systems (Figure 1A). In striking contrast, seedlings exposed to SQR9 VOCs were visibly healthier, larger, and maintained a greener phenotype. Quantitatively, the SQR9 VOCs significantly mitigated the effects of salt stress, leading to 141.4% greater total biomass (Figure 1B), 24.0% longer primary roots (Figure 1C), and 97.5% more lateral roots (Figure 1D) compared to the salt-stressed control group. These results clearly demonstrate that VOCs emitted by SQR9 confer robust, non-contact protection against salt-induced growth inhibition.

### 2.2. Mitigation of Physiological Damage and Oxidative Stress

**Figure 2:**
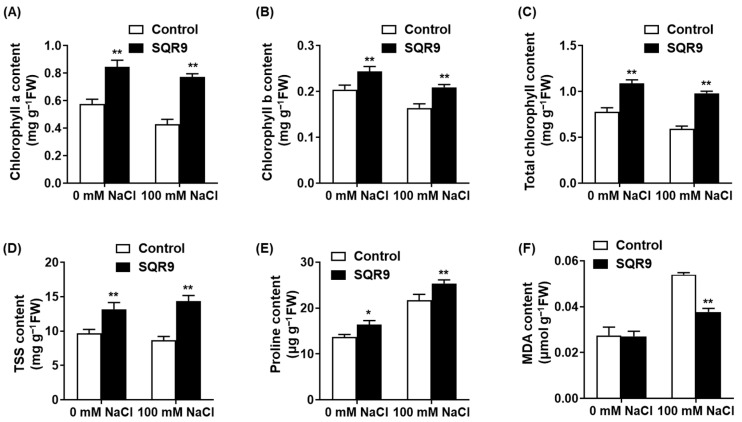
Effect of SQR9-emitted VOCs on chlorophyll, osmolytes, and MDA content in *Arabidopsis* under salt stress. The figure shows the quantification of key physiological indicators in *Arabidopsis* seedlings after 10 days of co-culture with SQR9 VOCs or sterile medium (Control), under 0 mM and 100 mM NaCl. (**A**) Average content of Chlorophyll *a* (mg g^−1^ FW). (**B**) Average content of Chlorophyll *b* (mg g^−1^ FW). (**C**) Average total chlorophyll content (mg g^−1^ FW). (**D**) Average content of total soluble sugars (TSS, mg g^−1^ FW). (**E**) Average content of proline (µg g^−1^ FW). (**F**) Average content of malondialdehyde (MDA, µmol g^−1^ FW). All data are presented as mean ± SD. Single asterisk (*) indicates a significant difference (*p* < 0.05), and double asterisks (**) indicate a highly significant difference (*p* < 0.01), compared to the Control group under the corresponding NaCl concentration.

We next assessed key physiological indicators to understand how SQR9 VOCs alleviated salt stress. As expected, exposure to 100 mM NaCl caused significant degradation of the photosynthetic machinery in control seedlings, resulting in decreases of 25.3%, 19.7%, and 23.8% in Chlorophyll a, Chlorophyll b, and total chlorophyll content, respectively (Figure 2A–C). Exposure to SQR9 VOCs significantly protected the chlorophyll, maintaining all three parameters at levels 27.5% to 79.8% higher than the stressed controls and statistically similar to the non-stressed controls.

We also measured the accumulation of protective osmolytes. Salt stress alone induced a predictable increase in both total soluble sugars (TSS) and proline (Figure 2D,E). Notably, SQR9 VOC exposure further enhanced this adaptive response; under 100 mM NaCl, VOC-treated seedlings accumulated 65.3% more TSS and 16.9% more proline than the stressed-control seedlings, suggesting a superior capacity for osmotic adjustment. To quantify cellular damage, we measured the content of malondialdehyde (MDA), a key product of lipid peroxidation. Salt stress triggered a 1.97-fold increase in MDA content in control seedlings (Figure 2F). SQR9 VOCs significantly counteracted this damage, reducing the MDA content by 30.1% compared to the stressed control group. This indicates a marked reduction in salt-induced oxidative damage to cell membranes.

### 2.3. Alleviation of ROS Accumulation via Antioxidant Enzymes

**Figure 3:**
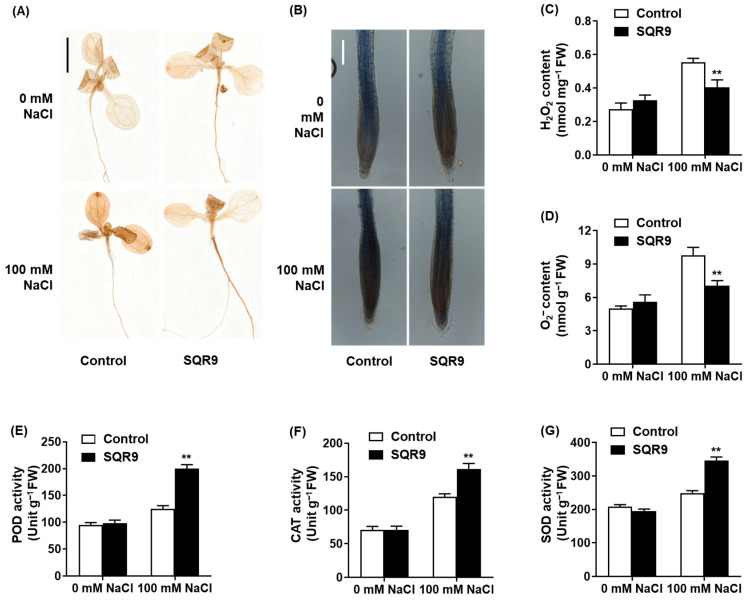
Effect of SQR9-emitted VOCs on ROS accumulation and antioxidant enzyme activities in *Arabidopsis*. The figure displays the accumulation and content of reactive oxygen species (ROS; H_2_O_2_ and O_2_^−^) and the activities of antioxidant enzymes in 10-day-old *Arabidopsis* seedlings co-cultured with SQR9 VOCs or sterile medium (Control), under 0 mM or 100 mM NaCl. (**A**) Histochemical detection of H_2_O_2_ in seedlings (DAB staining). Scale bar: 1 cm. (**B**) Histochemical detection of O_2_^−^ in roots (NBT staining). Scale bar: 100 µm. (**C**) Quantitative analysis of H2O2 content (nmol mg^−1^ FW). (**D**) Quantitative analysis of O_2_^−^ content (nmol g^−1^ FW). (**E**) Average peroxidase (POD) activity (Unit g^−1^ FW). (**F**) Average catalase (CAT) activity (Unit g^−1^ FW). (**G**) Average superoxide dismutase (SOD) activity (Unit g^−1^ FW). All quantitative data (**C**) to (**G**) are presented as mean ± SD. Double asterisks (**) indicate a highly significant difference (*p* < 0.01) compared to the Control group under the corresponding NaCl concentration.

The significant reduction in MDA content suggested that SQR9 VOCs modulate the plant’s ROS homeostasis. To confirm this, we performed histochemical staining for H_2_O_2_ (DAB) and O_2_^−^ (NBT). As shown in Figure 3A,B, 100 mM NaCl caused a massive accumulation of ROS, visualized as dark brown (DAB) and dark blue (NBT) precipitates throughout the seedlings, particularly in the roots. This accumulation was visibly abolished in the seedlings co-cultured with SQR9 VOCs, which resembled the non-stressed controls.

Quantitative analysis confirmed these visual observations. Compared to the stressed control group, SQR9 VOCs reduced the salt-induced accumulation of H_2_O_2_ by 27.0% (Figure 3C) and O_2_^−^ by 28.1% (Figure 3D). We hypothesized that this ROS reduction was due to an enhanced enzymatic scavenging capacity. Indeed, analysis of antioxidant enzyme activities revealed that SQR9 VOCs significantly “primed” the plant’s defenses. Under 100 mM NaCl, the activities of peroxidase (POD), catalase (CAT), and superoxide dismutase (SOD) in SQR9-exposed seedlings were 60.3%, 34.5%, and 39.2% higher, respectively, than in the stressed control seedlings (Figure 3E–G). This demonstrates that SQR9 VOCs actively enhance the plant’s enzymatic antioxidant defense system to neutralize salt-induced ROS.

### 2.4. Maintenance of Ion Homeostasis

**Figure 4:**
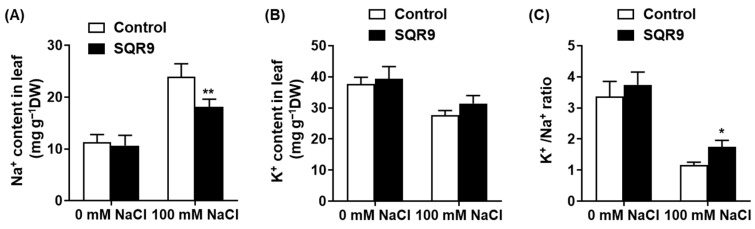
Effect of SQR9-emitted VOCs on ion homeostasis in *Arabidopsis* leaves. The figure displays the quantification of Na^+^ and K^+^ ion contents in the leaves of 10-day-old *Arabidopsis* seedlings co-cultured with SQR9 VOCs or sterile medium (Control), under 0 mM or 100 mM NaCl. (**A**) Average Na^+^ content (mg g^−1^ Dry Weight, DW). (**B**) Average K^+^ content (mg g^−1^ DW). (**C**) The corresponding K^+^/Na^+^ ratio calculated from (**A**,**B**). All data are presented as mean ± SD. Single asterisk (*) or double asterisks (**) indicate a significant (*p* < 0.05) or highly significant (*p* < 0.01) difference, respectively, compared to the Control group under the corresponding NaCl concentration.

To assess the second pillar of salt tolerance, we investigated whether SQR9 VOCs could mitigate the ionic toxicity component of salt stress. We measured the Na^+^ and K^+^ content in the leaves of seedlings after 10 days of treatment (Figure 4). Under non-saline conditions, no significant differences in ion content were observed (Figure 4A,B). However, exposure to 100 mM NaCl caused a massive and toxic accumulation of Na^+^ in the leaves of control seedlings, reaching 24.0 mg g^−1^ dry weight (DW). Remarkably, co-culture with SQR9 VOCs significantly suppressed this influx; Na^+^ accumulation in VOC-treated seedlings was reduced by 24.5% to 18.1 mg g^−1^, a level highly significant compared to the stressed-control group (Figure 4A).

Conversely, salt stress caused a substantial loss of the essential nutrient K^+^ in control seedlings. SQR9 VOC exposure helped seedlings maintain a higher K^+^ content (31.5 mg g^−1^ DW) compared to the stressed controls (27.7 mg g^−1^ DW), although this difference was not statistically significant (Figure 4B). However, the strong and significant reduction in toxic Na^+^ accumulation, combined with the trend of K^+^ retention, resulted in a statistically significant improvement in the K^+^/Na^+^ ratio. This critical metric of salt tolerance collapsed to ~1.2 in stressed controls, but was maintained at ~1.7—a 50.6% increase—in the SQR9 VOC-treated seedlings (Figure 4C). Together, these data demonstrate that SQR9 VOCs effectively protect plants from salt-induced ionic toxicity, primarily by modulating ion transport to reduce Na^+^ uptake/accumulation and maintain a more favorable K^+^/Na^+^ ratio.

### 2.5. Transcriptional Regulation of Stress-Related Genes

**Figure 5:**
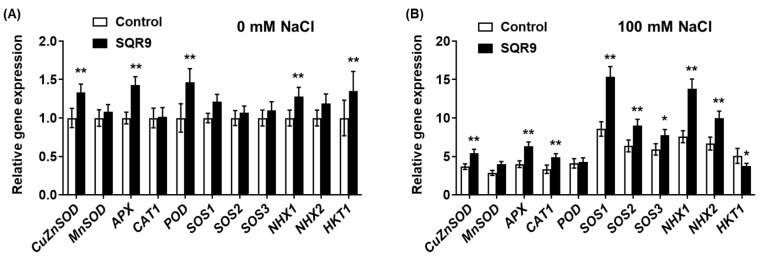
Effect of SQR9-emitted VOCs on the relative expression levels of salt-stress-related genes in *Arabidopsis*. The figure displays the relative gene expression levels of salt-stress-related genes in *Arabidopsis* seedlings after 4 days of co-culture with SQR9 VOCs or sterile medium (Control). (**A**) Relative gene expression under non-saline conditions (0 mM NaCl). (**B**) Relative gene expression under salt-stressed conditions (100 mM NaCl). The analyzed genes include ROS-scavenging enzyme genes (*CuZnSOD*, *MnSOD*, *APX*, *CAT1*, *POD*) and ion homeostasis-related genes (*SOS1*, *SOS2*, *SOS3*, *NHX1*, *NHX2*, *HKT1*). The *EEF1a4* gene was used as the internal control. All data are presented as mean ± SD. Single asterisk (*) or double asterisks (**) indicate a significant (*p* < 0.05) or highly significant (*p* < 0.01) difference, respectively, compared to the Control group under the corresponding NaCl concentration.

To elucidate the molecular mechanisms underlying the enhanced enzyme activities (Figure 3) and improved ion homeostasis (Figure 4), we performed qRT-PCR analysis on seedlings co-cultured for 4 days. Under non-saline conditions (0 mM NaCl), exposure to SQR9 VOCs alone was sufficient to induce a “priming” state, characterized by the modest but significant upregulation of several genes (Figure 5A). This included the ROS-scavenging genes *CuZnSOD*, *APX*, and *POD*, as well as the ion transporters *NHX1* and *HKT1*. Notably, under these normal conditions, *MnSOD*, *CAT1*, the core *SOS* pathway genes (*SOS1*, *SOS2*, *SOS3*), and *NHX2* were not significantly affected.

This priming effect was dramatically amplified and strategically re-patterned upon the application of salt stress (100 mM NaCl) (Figure 5B). Compared to the stressed-control group, SQR9-exposed seedlings exhibited a strong and coordinated transcriptional upregulation of key ROS-scavenging enzyme genes, including *CuZnSOD*, *APX*, and *CAT1*. In sharp contrast to the 0 mM results, the *POD* gene was not significantly upregulated under salt stress, nor was *MnSOD*. This specific upregulation pattern suggests that SQR9 VOC-mediated priming predominantly targets the cytosolic, chloroplastic, and peroxisomal (*CuZnSOD*, *APX*, *CAT1*) antioxidant pathways rather than the mitochondrial pathway (*MnSOD*) under salt stress.

In striking parallel, SQR9 VOCs simultaneously orchestrated the complete and powerful upregulation of the ion homeostasis network. The key *SOS* pathway genes—*SOS1*, *SOS2*, and *SOS3*—were all strongly upregulated. Concurrently, the vacuolar sequestration genes *NHX1* and *NHX2* were also significantly upregulated. Most strikingly, the *HKT1* gene showed an opposite regulation. While *HKT1* was upregulated by VOCs under control conditions (Figure 5A), it was significantly downregulated by 26.0% in SQR9-treated seedlings under salt stress (Figure 5B). This comprehensive, dual-action regulation of ion transporters—simultaneously upregulating efflux/sequestration (via *SOS*/*NHX*) and downregulating influx/recirculation (via *HKT1*)—provides a direct and sophisticated molecular basis for the salt-tolerant phenotype.

### 2.6. Validation of 2,3-Butanediol as the Active Compound

**Figure 6:**
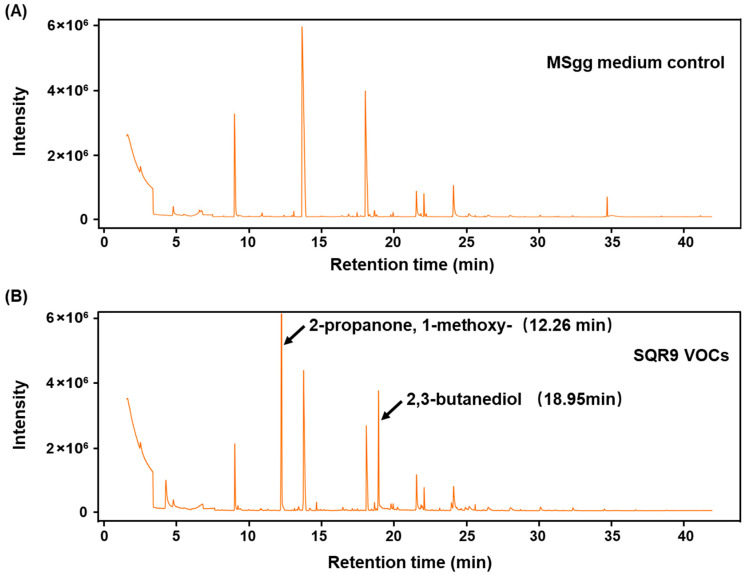
Gas chromatography–mass spectrometry (GC-MS) analysis of VOCs emitted by *B. velezensis* SQR9. The figure shows the GC-MS chromatograms of VOCs from the SQR9 strain culture compared to the control medium. SQR9 was cultured on MSgg solid medium for 7 days for VOCs collection. (**A**) GC-MS chromatogram of the sterile MSgg medium control. (**B**) GC-MS chromatogram of VOCs collected from the SQR9 culture. Peaks corresponding to 2-propanone, 1-methoxy (RT ~12.26 min) and 2,3-butanediol (RT ~18.95 min) are labeled.

To identify the specific active compound(s) responsible for the observed effects, the headspace of SQR9 (cultured on MSgg medium) was collected by SPME and analyzed by GC-MS. The resulting chromatogram (Figure 6B) revealed a complex blend of SQR9-specific VOCs that were absent in the sterile medium control (Figure 6A). The top ten most abundant compounds were identified and are listed in Supplementary Table S2. Among these, two compounds, 2,3-butanediol (2,3-BD) (retention time [RT], ~18.95 min) and 2-propanone, 1-methoxy (2-P, 1-M) (retention time [RT], ~12.26 min), were consistently detected as major, distinct peaks in the SQR9 culture and were selected for functional validation.

**Figure 7:**
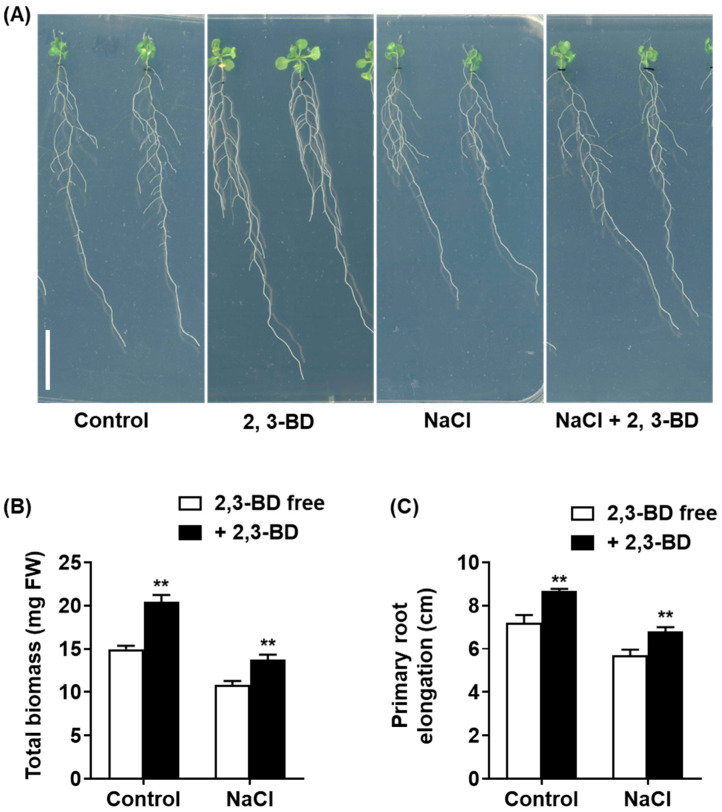
Exogenous application of 2,3-butanediol (2,3-BD) promotes growth in *Arabidopsis* under non-saline and salt-stressed conditions. The figure displays the effect of synthetic 2,3-BD (100 µM) exposure on 4-day-old *Arabidopsis* seedlings grown for 10 days on 1/2 MS medium with or without 100 mM NaCl. (**A**) Phenotypic analysis of seedlings. Scale bar: 1 cm. (**B**) Average total biomass (mg FW). (**C**) Average primary root elongation (cm). All quantitative data are presented as mean ± SD. Double asterisks (**) indicate a highly significant difference (*p* < 0.01) compared to the respective 2,3-BD -free group (Control or NaCl-only treated).

We first tested the bioactivity of synthetic 2,3-BD using a dose–response assay under non-saline conditions (Supplementary Figure S1). Exposure to 2,3-BD VOCs exhibited a clear, dose-dependent growth-promoting effect on Arabidopsis seedlings (Figure S1A). Significant increases in total biomass (Figure S1B) and primary root elongation (Figure S1C) were observed at concentrations from 10 µM to 300 µM, with an optimal effect observed at approximately 100 µM. We then used an optimal concentration (100 µM) to test whether 2,3-BD could mimic the salt-protective effects of the SQR9 bacterial culture. As shown in Figure 7, the application of synthetic 2,3-BD VOCs successfully replicated the phenotype. Under 100 mM NaCl, 2,3-BD-exposed seedlings were visibly larger and healthier (Figure 7A) and possessed 26.8% more total biomass (Figure 7B) and 19.3% greater primary root elongation (Figure 7C) than the NaCl-only controls. In contrast, the other identified compound, 2-P, 1-M, was tested across the same concentration range and showed no growth-promoting or salt-protective effects whatsoever (Supplementary Figure S2). These validation experiments strongly identify 2,3-butanediol as the key active volatile effector molecule responsible for the growth promotion and salt tolerance mediated by SQR9 VOCs.

## 3. Discussion

The study demonstrates that *Bacillus velezensis* SQR9, through a volatile-mediated pathway, can systemically enhance the salt tolerance of *Arabidopsis thaliana*. This protective effect was comprehensive, alleviating salt-induced growth inhibition, mitigating physiological damage to photosynthetic pigments, and reducing cellular oxidative damage. Crucially, we identified 2,3-BD as the key volatile effector molecule.

We identified the priming of antioxidant defense as a key mechanism, essential for managing ROS under salinity [39]. SQR9 VOCs mitigated oxidative stress by reducing H_2_O_2_ and O_2_^−^ levels via enhanced SOD, CAT, and POD activities (Figure 3). Transcriptional profiling indicates a nuanced regulatory network: *CAT1* and *APX* were strongly upregulated, directly correlating with observed enzymatic boosts. In contrast, total SOD activity was likely sustained by the upregulation of cytosolic/chloroplastic *CuZnSOD* rather than mitochondrial *MnSOD* [40]. Additionally, the disconnect between elevated POD activity and the lack of stress-induced gene upregulation suggests involvement of post-transcriptional regulation or other isoenzymes. Thus, SQR9 VOCs orchestrate a robust, multi-pathway response, primarily anchored by the transcriptional activation of the *CuZnSOD*/*APX*/*CAT1* pathway.

Simultaneously, SQR9 VOCs maintain plant ion homeostasis by significantly reducing toxic Na^+^ accumulation and boosting the K^+^/Na^+^ ratio, effectively countering the primary driver of salt stress [41]. This protection relies on a “push–pull” mechanism: the coordinated transcriptional upregulation of the SOS pathway (*SOS1*, *SOS2*, *SOS3*) for Na^+^ efflux [42,43] and *NHX1*/*NHX2* for vacuolar sequestration [44,45]. Notably, while the PGPR strain *Bacillus subtilis* GB03 confers salt tolerance via *HKT1*-mediated Na^+^ recirculation independent of the SOS pathway [46], SQR9-treated plants exhibit *HKT1* downregulation alongside strong *SOS1* induction. We propose that SQR9 mediates a “prevention-first” strategy where active exclusion via *SOS1* minimizes the need for *HKT1*-based retrieval. These findings demonstrate that distinct PGPR strains modulate ion homeostasis through divergent pathways—exclusion (SQR9) versus recirculation (GB03)—to prevent shoot Na^+^ overaccumulation.

Our study identified 2,3-BD as the predominant bioactive volatile emitted by SQR9. 2,3-BD is a well-established volatile produced by PGPR, widely recognized for its ability to promote plant growth [47], induce systemic resistance (ISR) against pathogens [48,49], enhance drought tolerance [50,51], and reduce disease incidence in crops [52]. Our validation experiments substantiated its role in alleviating salt stress; notably, synthetic 2,3-BD successfully recapitulated both the growth-promoting and salt-protective effects observed in the SQR9 co-culture. These findings establish 2,3-BD as the primary active compound in this system, supporting previous reports that characterize 2,3-BD as a broad-spectrum mitigator of abiotic stress.

Notably, a previous study attributed SQR9-mediated salt tolerance solely to the non-volatile polyamine, spermidine, and reported VOCs to be ineffective [32]. Rather than a contradiction, this discrepancy highlights the metabolic plasticity of SQR9. We propose that this divergence is largely attributable to critical differences in experimental methodology. First, the 2017 study cultured SQR9 on 1/2 MS medium, a minimal salts medium designed for plants. In contrast, our study used MSgg medium, which is rich in carbon (glycerol) and nitrogen (glutamate) sources. 2,3-butanediol is a classic end-product of mixed-acid fermentation, a pathway that is heavily favored under carbon-rich conditions [53]. The nutrient-poor 1/2 MS medium likely failed to induce the metabolic pathway for 2,3-BD production, whereas the MSgg medium strongly promoted it. Moreover, the robust production of 2,3-BD by SQR9 observed in this study is consistent with its genomic features. The SQR9 genome harbors the conserved *alsRSD* operon, which is responsible for the metabolic flux from pyruvate to 2,3-BD [37]. Second, our protocol included a 3-day bacterial pre-culture, which allowed 2,3-BD to accumulate to an effective concentration in the plate before the plants were introduced. Thus, our study does not contradict the 2017 findings but rather complements them. SQR9 possesses at least two distinct, condition-dependent effector pathways (non-volatile spermidine and volatile 2,3-BD) to achieve the same outcome. Significantly, both spermidine and 2,3-BD converge on the exact same plant defense systems: activation of the antioxidant machinery and activation of the SOS/NHX ion homeostasis pathways. This convergence suggests these two plant pathways are the primary targets for SQR9, which has evolved redundant effector strategies to ensure their activation.

These findings highlight that PGPR, such as the *B. velezensis* SQR9, are increasingly recognized as a category of microbial plant biostimulants. These beneficial microorganisms enhance plant tolerance to abiotic stresses (e.g., salinity, drought) through mechanisms analogous to those defined for traditional biostimulants, such as improved nutrient uptake, modulation of phytohormone signaling, activation of antioxidant defense systems, and maintenance of ion homeostasis. By inducing systemic priming and conferring non-contact stress protection via volatiles like 2,3-butanediol, PGPR offer promising, eco-friendly alternatives for improving crop resilience in saline or otherwise stressed agricultural soils.

While our study offers detailed mechanistic insights using *Arabidopsis thaliana* in controlled in vitro split-plate systems, we acknowledge limitations in extrapolating these findings to field conditions. The closed system maximizes VOC accumulation, whereas in open agricultural environments, soil porosity and atmospheric diffusion may dilute volatile concentrations and reduce efficacy. Moreover, *Arabidopsis* responses may not fully reflect those of crop species exposed to complex interactions with native soil microbiomes and dynamic environmental stresses. Thus, practical application of SQR9-derived VOCs—particularly 2,3-butanediol—will require optimized delivery strategies (e.g., soil drenching or encapsulation) and validation through multi-year field trials to ensure robust, scalable performance under real-world conditions.

## 4. Materials and Methods

### 4.1. Plant Material and Bacterial Strain

*Arabidopsis thaliana* (L.) Heynh. ecotype Columbia-0 (Col-0) was used as the wild-type plant material for all experiments. The bacterial strain *Bacillus velezensis* SQR9 (CCTCC No. 5808), previously isolated from cucumber rhizosphere [54], was used for all bacterial–plant interaction assays.

### 4.2. Bacterial Culture and Plant Growth Conditions

*B. velezensis* SQR9 was streaked from a −80 °C glycerol stock onto a Luria–Bertani (LB) solid plate for activation. A single colony was then inoculated into LB liquid medium and cultured at 37 °C with 170 rpm shaking for 24 h. The bacterial culture was centrifuged (5000 rpm, 3 min), washed three times with sterile distilled water, and finally resuspended in sterile water to a final optical density (OD_600_) of 1.0. For plant growth, *Arabidopsis* Col-0 seeds were surface-sterilized with 1% (*v*/*v*) sodium hypochlorite (NaClO) for 8 min, washed 4–5 times with sterile water, and stratified at 4 °C in the dark for 2–3 days. Sterilized seeds were sown on 1/2 MS salts solid medium [2.15 g L^−1^ MS salts (PhytoTech Labs, Lenexa, KS, USA), 0.1 g L^−1^ myo-inositol, 0.5 g L^−1^ MES, 10 g L^−1^ sucrose, and 10 g L^−1^ agar (Solarbio, Beijing, China), pH 5.7 adjusted with KOH]. Plates were placed vertically in a growth chamber (22 °C, 16 h light/8 h dark photoperiod, 100 µmol m^−2^ s^−1^) for 4 days. Four-day-old seedlings with uniform growth were selected for subsequent experiments.

### 4.3. Plant–Bacterial VOC Co-Culture Assay

The volatile organic compound (VOC) co-culture assay was performed in 9 cm two-compartment Petri dishes (Supplementary Figure S3). One compartment was filled with 1/2 MS salts solid medium, supplemented with either 0 mM or 100 mM NaCl. The other compartment was filled with MSgg (minimal salts glycerol glutamate) solid medium. A 3 µL aliquot of the resuspended SQR9 bacterial suspension (OD_600_ = 1.0) or sterile water (as a control) was spotted onto the MSgg medium. These plates were incubated at 30 °C for 3 days to allow for bacterial pre-culture and VOC production. After the pre-incubation, 4-day-old *Arabidopsis* seedlings were transferred onto the 1/2 MS medium in the adjacent compartment. The plates were immediately sealed with Parafilm (Bemis, Neenah, WI, USA) and returned to the 22 °C growth chamber. Seedlings were co-cultured for 4 days for qRT-PCR analysis or for 10 days for all phenotypic, physiological, and ion content analyses.

### 4.4. Phenotypic Analysis

At the end of the 10-day co-culture period, the entire sealed split-plates were scanned (EPSON XL11000, Tokyo, Japan). Primary root elongation was measured from the scanned images using Fiji software (ImageJ, v1.53). The number of lateral roots that emerged was counted manually. The total fresh weight (FW) of individual seedlings was measured immediately using an analytical balance.

### 4.5. Measurement of Physiological and Biochemical Parameters

For all biochemical assays, plant tissues were harvested after the 10-day co-culture treatment and immediately frozen in liquid nitrogen.

#### 4.5.1. Chlorophyll Content

Frozen tissue (0.1 g) was ground in liquid nitrogen and 1 mL of 80% (*v*/*v*) acetone was added. The mixture was vortexed and centrifuged at 13,000× *g* for 10 min at 4 °C. The supernatant’s absorbance was measured at 663.6 nm and 646.6 nm. Chlorophyll a (Chl a), Chlorophyll b (Chl b), and total chlorophyll content were calculated using the equations described by Porra (2002) [55].

#### 4.5.2. Total Soluble Sugar (TSS) Content

TSS content was determined using the anthrone–sulfuric acid method [56]. 0.1 g of ground tissue was boiled in 10 mL sterile water for 1 h. 0.1 g of activated charcoal was added, and the mixture was boiled again for 30 min. After centrifugation (13,000× *g*, 10 min), 200 µL of the supernatant was mixed with 1 mL of 0.2% anthrone reagent, boiled for 30 min, and cooled. Absorbance was read at 620 nm. TSS content was quantified using a sucrose standard curve.

#### 4.5.3. Proline Content

Proline content was measured using the acid-ninhydrin method [57]. 0.1 g of ground tissue was homogenized in 1 mL of 3% (*w*/*v*) sulfosalicylic acid and centrifuged (13,000× *g*, 10 min). 200 µL of the supernatant was mixed with 500 µL of glacial acetic acid and 500 µL of acidic ninhydrin, then boiled for 30 min. The reaction was stopped in an ice bath, and absorbance was read at 520 nm. Proline content was quantified using an L-proline standard curve.

#### 4.5.4. Malondialdehyde (MDA) Content

Lipid peroxidation was estimated by measuring MDA content using the modified thiobarbituric acid (TBA) method [58]. Absorbance was measured at 450 nm, 532 nm, and 600 nm. MDA concentration (µmol g^−1^ FW) was calculated using the formula: [MDA] = 6.45 (A_532_ − A_600_) − 0.56 A_450_.

### 4.6. Histochemical Staining and ROS-Related Assays

Histochemical staining was performed on seedlings after the 10-day co-culture according to the methods described by Fryer et al. (2002) [59]. For H_2_O_2_ detection, entire seedlings were submerged in 0.1% (*w*/*v*) 3,3′-diaminobenzidine (DAB) solution and incubated at 28 °C in the dark for 12 h. For O_2_^−^ detection, seedling roots were submerged in 1 g L^−1^ nitroblue tetrazolium (NBT) in 10 mM phosphate buffer (pH 7.8) and incubated at 28 °C in the dark for 4 h. Seedling images after histochemical staining were captured using a Leica DM2500 microscope (Leica Microsystems, Wetzlar, Germany) equipped with a digital camera. The quantitative contents of H_2_O_2_ and O_2_^−^, as well as the activities of antioxidant enzymes (Catalase, CAT; Peroxidase, POD; Superoxide Dismutase, SOD), were determined using commercial detection kits (Suzhou Comin Biotechnology Co., Ltd., Suzhou, China) according to the manufacturer’s instructions. Absorbance measurements for biochemical assays were conducted on a SpectraMax M5 microplate reader (Molecular Devices, San Jose, CA, USA).

### 4.7. Ion Content Measurement

Shoots (leaves and stems) from seedling samples (harvested after 10-day co-culture) were dried at 105 °C for 30 min and then at 65 °C for 3 days to a constant weight. 0.1 g of the dried powder was digested with concentrated HNO_3_ and H_2_O_2_ at 220 °C in a muffle furnace for 60 min. The concentrations of Na^+^ and K^+^ in the digested solution were determined using a flame photometer (M410; Sherwood Scientific Ltd., Cambridge, UK). The K^+^/Na^+^ ratio was calculated by dividing the K^+^ content by the Na^+^ content for each sample.

### 4.8. VOC Analysis by SPME-GC-MS

SQR9 was cultured on MSgg solid medium in a 20 mL headspace vial for 7 days. Volatiles were extracted using a solid-phase microextraction (SPME) fiber (50/30 µm DVB/CAR/PDMS; Supelco, Bellefonte, PA, USA) at 40 °C for 60 min with agitation. GC-MS analysis was performed on an Agilent 7890A chromatograph with 5975C mass selective detector (MSD) (Agilent Technologies, Santa Clara, CA, USA) equipped with a DB-WAX capillary column (30 m × 0.25 mm × 0.25 µm). The oven temperature was programmed as follows: 40 °C for 3 min, ramped at 4 °C min^−1^ to 220 °C, and held for 10 min. Helium was used as the carrier gas (1.0 mL min^−1^). Mass spectra were obtained in EI mode (70 eV) with a scanning range of *m*/*z* 20–650. Compounds were tentatively identified by matching their mass spectra with the NIST08 library (match value > 70). Data were normalized using the total peak area.

### 4.9. Synthetic VOC Treatment

Synthetic 2,3-butanediol (2,3-BD) and 2-propanone, 1-methoxy (2-P, 1-M) were purchased from Sigma-Aldrich (St. Louis, MO, USA). Compounds were dissolved in DMSO to create stock solutions, which were then serially diluted in DMSO to the final test concentrations. For the assay, 50 µL of the compound solution (or 50 µL pure DMSO as a control) was applied to a sterile filter paper disc. The disc was placed in one compartment of a 9 cm split-plate. Four-day-old Arabidopsis seedlings were placed on the other compartment (1/2 MS medium with or without 100 mM NaCl). Plates were sealed with Parafilm and incubated at 22 °C for 10 days. After incubation, seedlings were scanned (EPSON XL11000) for phenotypic analysis.

### 4.10. Quantitative Real-Time PCR (qRT-PCR)

Total RNA was extracted from the whole seedlings (co-cultured for 4 days) using a plant RNA isolation kit (OMEGA Bio-tek, Norcross, GA, USA). First-strand cDNA was synthesized using the HiScript II First Strand cDNA Synthesis Kit (Vazyme, Nanjing, China). qRT-PCR was performed on a StepOnePlus™ Real-Time PCR System (Applied Biosystems, Foster City, CA, USA) using ChamQ SYBR Color qPCR Master Mix (Vazyme). The thermal cycling conditions were: 95 °C for 30 s, followed by 40 cycles of 95 °C for 10 s and 60 °C for 30 s. The *Arabidopsis* gene *EEF1a4* (AT5G60390) was used as the internal reference gene for normalization. The relative expression levels of target genes were calculated using the 2^−^ΔΔCt method [60]. All primer sequences used in this study are listed in Supplementary Table S1.

### 4.11. Statistical Analysis

All experiments were performed with at least three independent biological replicates. Data are presented as the mean ± standard deviation (SD). Statistical analyses were performed using GraphPad Prism 9 (GraphPad Software, San Diego, CA, USA). Two-group comparisons were analyzed using a two-tailed Student’s *t*-test. Multi-group comparisons were analyzed using a one-way analysis of variance (ANOVA) followed by Tukey’s post hoc test. Differences were considered statistically significant at *p* < 0.05 (*) and highly significant at *p* < 0.01 (**).

## 5. Conclusions

**Figure 8:**
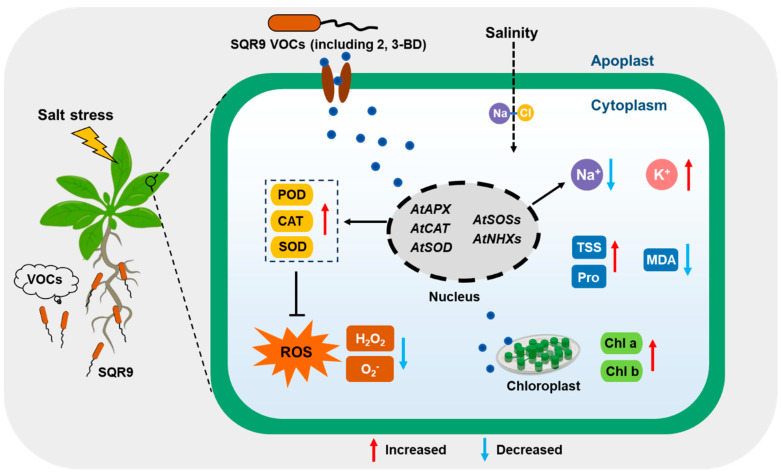
A proposed working model for B. velezensis SQR9-emitted VOCs-mediated salt tolerance in *Arabidopsis thaliana*. Under salt stress, SQR9 releases volatile organic compounds (VOCs), including the key effector 2,3-butanediol (2,3-BD). Perception of these VOCs triggers a signaling cascade that transcriptionally upregulates ROS-scavenging genes (*AtAPX*, *AtCAT*, *AtSOD*) and ion homeostasis genes (*AtSOSs*, *AtNHXs*), while downregulating *AtHKT1*. This dual regulation enhances antioxidant enzyme activities (POD/CAT/SOD) to detoxify ROS and optimizes ion transport (Na^+^ efflux/sequestration and K^+^ retention) to maintain a high K^+^/Na^+^ ratio. Consequently, SQR9 VOCs mitigate oxidative damage and protect photosynthetic machinery, promoting plant growth and salt tolerance. Red arrows indicate upregulation or increase; blue arrows indicate downregulation or decrease.

In conclusion, this study demonstrates that VOCs emitted by *Bacillus velezensis* SQR9 provide robust, non-contact protection to *Arabidopsis thaliana* against salt stress (Figure 8). Crucially, we identified 2,3-BD as the key active volatile responsible for this effect. Mechanistically, we propose that SQR9 VOCs act as a systemic priming signal, initiating a dual-pronged defense strategy: (1) co-activating the antioxidant enzyme system (via the upregulation of *CuZnSOD*, *APX*, and *CAT1*) to neutralize the ROS burst and reduce cellular oxidative damage; and (2) optimizing ion homeostasis (via the upregulation of *SOS* and *NHX* pathways, and downregulation of *HKT1*) to facilitate Na^+^ efflux/sequestration and maintain a high K^+^/Na^+^ ratio. This coordinated regulation protects the photosynthetic machinery and sustains plant growth under high salinity. These findings expand the understanding of SQR9’s condition-dependent metabolic strategies and highlight 2,3-butanediol as a promising microbial biostimulant for developing climate-resilient crops.

## Data Availability

The original contributions presented in this study are included in the article/Supplementary Material. Further inquiries can be directed to the corresponding author.

## Supplementary Materials

The following supporting information can be downloaded at: https://www.mdpi.com/article/10.3390/plants15020218/s1, Figure S1: Dose-dependent effect of 2,3-butanediol (2,3-BD) on the growth of *Arabidopsis* seedlings. Figure S2: Effect of exogenous 2-propanone, 1-methoxy (2-P, 1-M) on the growth of *Arabidopsis* seedlings. Figure S3. Schematic illustration of the split-plate co-culture system for bacteria and plants. Table S1: Primer sequences used for qRT-PCR in this study. Table S2: Top ten VOCs identified from *B. velezensis* SQR9 by GC-MS analysis.

## Abbreviations

The following abbreviations are used in this manuscript:

2,3-BD	2,3-Butanediol
CAT	Catalase
GC-MS	Gas Chromatography–Mass Spectrometry
HKT	High-affinity K^+^ Transporter
MDA	Malondialdehyde
NHX	Na^+^/H^+^ Exchanger
PGPR	Plant Growth-Promoting Rhizobacteria
POD	Peroxidase
qRT-PCR	Quantitative Real-Time Polymerase Chain Reaction
ROS	Reactive Oxygen Species
SOD	Superoxide Dismutase
SOS	Salt Overly Sensitive
VOCs	Volatile Organic Compounds
