# Supplementary material for: Bacillus velezensis SQR9-Emitted Volatiles Enhance Arabidopsis Salt Tolerance via ROS Scavenging and Ion Transport Regulation
Source: Plants (Basel). 2026 Jan 10;15(2):218. doi: 10.3390/plants15020218 (PMC12845509; doi:10.3390/plants15020218)
Supplement: Supplementary file 1 [file plants-15-00218-s001.zip › plants-4047429-supplementary.pdf]

Supplementary Materials:

Figure S1: Dose-dependent effect of 2,3-butanediol (2,3-BD) on the growth of *Arabidopsis* seedlings.

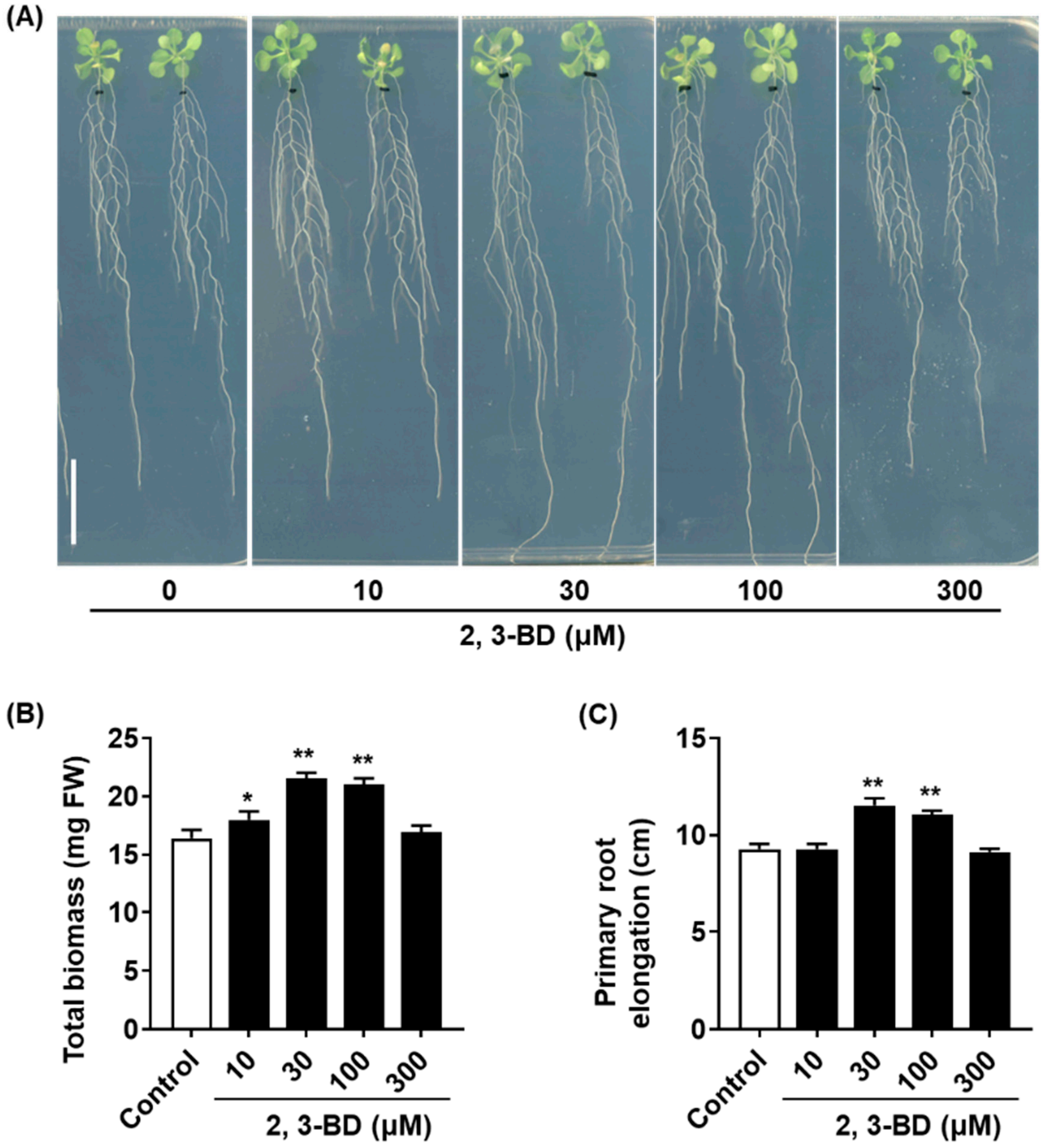

The effect of exposure to different concentrations of exogenous 2,3-BD VOCs (0, 10, 30, 100, and 300 μM) on 4-day-old *Arabidopsis* seedlings grown for 10 days on 1/2 MS medium. (A) Phenotypic analysis of seedlings. Scale bar: 1 cm. (B) Average total biomass (mg FW). (C) Average primary root elongation (cm). All quantitative data are presented as mean ± SD. Double asterisks (**) indicate a highly significant difference ($P < 0.01$) compared to the 0 μM Control group.

Figure S2: Effect of exogenous 2-propanone, 1-methoxy (2-P, 1-M) on the growth of *Arabidopsis* seedlings.

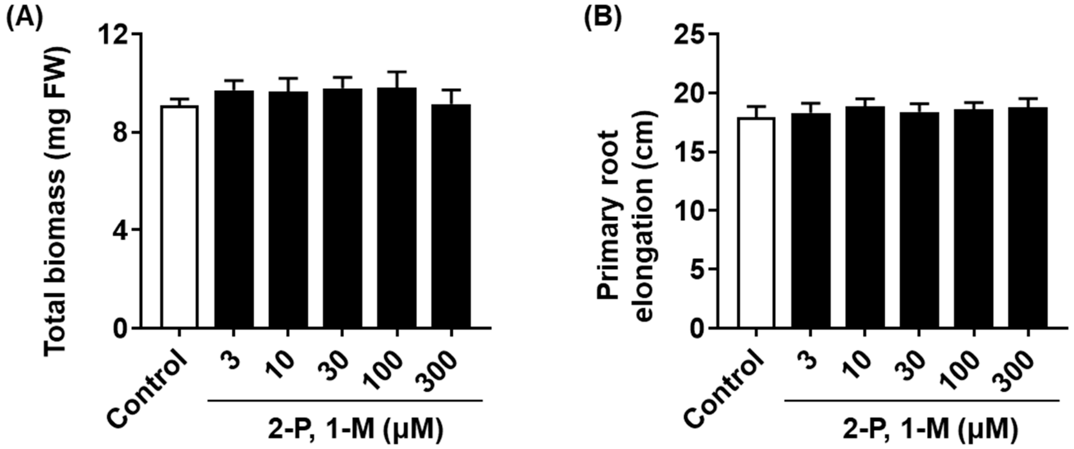

The growth parameters of *Arabidopsis* seedlings grown for 10 days on 1/2 MS medium and exposed to 2-P, 1-M VOCs at various concentrations (0, 3, 10, 30, 100, and 300 μM). (A) Average total biomass (mg FW). (B) Average primary root elongation (cm). All data are presented as mean ± SD. No significant differences were observed compared to the 0 μM Control group.

Figure S3. Schematic illustration of the split-plate co-culture system for bacteria and plants.

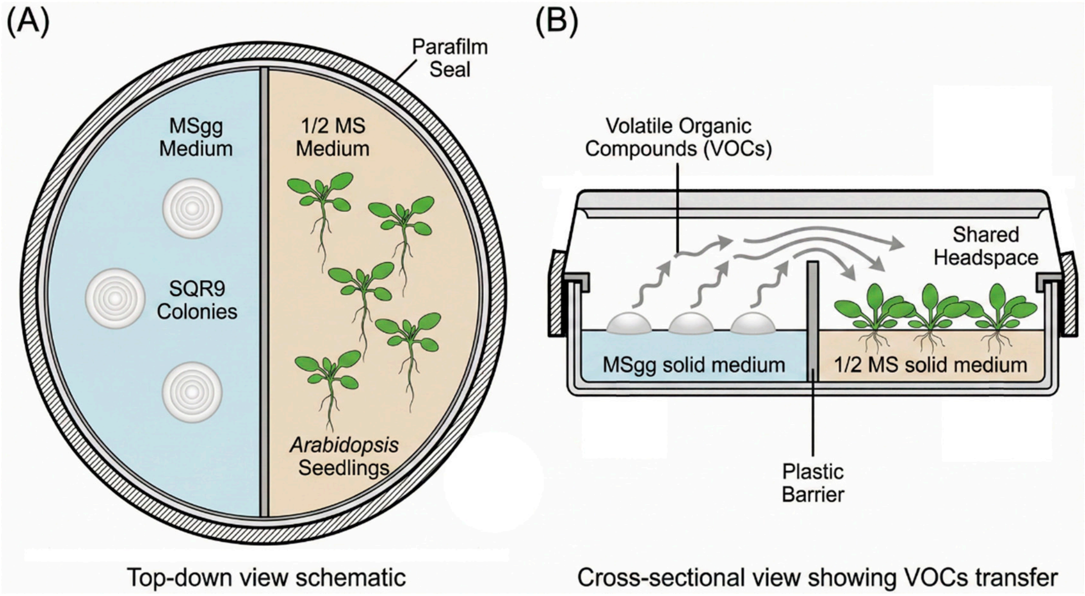

The setup is used to assess the effects of volatile organic compounds (VOCs) produced by bacteria (e.g., SQR9) on plant (*Arabidopsis*) growth. (A) Top-down schematic view of the split plate. (B) Cross-sectional side view illustrating the mechanism of VOC-mediated interaction.

Table S1: Primer sequences used for qRT-PCR in this study.

**Gene**	**Sequence 5'- 3'**
AtMn-SOD	F: CGCATGATCCTITGGCTTCG<br>R: TCCTGGTTGGCTGTGGTITC
AtCuZn-SOD	F: CTGCATCTCTACTGGACCTC<br>R: CCACATCCAACTCTCGAGC
AtAPX1	F: CTCTGGGACGATGCCACAAG<br>R: CTCGACCAAAGGACGGAAAA
AtCAT1	F: TCCTGTTATCGTTCGTTTCTCA<br>R: CAAAGTTCCCCTCTCTGGTGTA
AtPOD	F: CCAAACTCTTCGTGGACTATGC<br>R: AACTCTTGGTCGCTCTGGAT
AtSOS1	F: GATTTGTCCCCACGAATGAGA<br>R: TGCGAAGAAGGCGTAGAACA
AtSOS2	F: GCAAGGGAAGAAGAAGAAGT<br>R: TCTCCGCTACATAACTGCC
AtSOS3	F: GAATCCATCGCTCATCAA<br>R: CCATTTCTTCCTCTTCACA
AtNHX1	F: ATTGAGCCTTCAGGGAACCA<br>R: AAAGCCACGACCTCCAAAGA
AtNHX2	F: ATGACAATGTTCGCCTCTTTAACC<br>R: TCAAGGTTTACTAAGATCATGGCTG
AtHKT1	F: GATTTGTCCCCACGAATGAGA<br>R: CAAAACCAAGAAGCAAGGGAAC
AtEEF1a4	F: CTGGAGGTTTTGAGGCTGGTAT<br>R: CCAAGGGTGAAAGCAAGAAGA

Table S2: Top ten VOCs identified from *B. velezensis* SQR9 by GC-MS analysis.

**Average Rt(min)**	**Metabolite name**	**Normalized amount of volatile compound (%)**
12.259	2-propanone, 1-methoxy-	61.43
18.948	2,3-butanediol	26.40
24.001	2,5-hexanedione, 3,4-dihydroxy-3,4-dimethyl-	1.62
14.694	Butanoic acid, 2-methyl-3-oxo-, methyl ester	1.27
9.261	2-heptanone	0.96
13.778	Propanoic acid, 2-hydroxy-2-methyl-	0.67
24.953	2,6-dimethylbenzaldehyde	0.65
13.452	Pyrazine, 2,6-dimethyl-	0.61
7.65	3-hexanone, 2-methyl-	0.50
14.233	2-hydroxy-3-pentanone	0.43

The top ten most abundant volatile organic compounds (VOCs) detected in the headspace of *B. velezensis* SQR9 culture (as shown in Figure 6), identified based on their retention time (RT) and mass spectral matching. Columns show the average RT (min), metabolite name, and the normalized amount of the volatile compound (%), representing the percentage of the volatile compound's peak area relative to the total peak area of all identified VOCs.
